# Cannabis and Wound Healing: A Narrative Review of Current Evidence and Applications to Facial Plastic Surgery

**DOI:** 10.3390/jpm16090442

**Published:** 2026-08-24

**Authors:** Bita Rashed Naimi, David B. Hom

**Affiliations:** Department of Otolaryngology–Head and Neck Surgery, University of California San Diego Health, San Diego, CA 92103, USA; bnaimi@health.ucsd.edu

**Keywords:** cannabis, marijuana, facial plastic surgery, wound healing, postoperative outcomes, complications, infection, hematoma, facial trauma, mandibular fracture, precision medicine

## Abstract

Cannabis use has increased substantially in the United States, driven by broader legalization, decriminalization, and expanding medical and recreational availability. For facial plastic surgeons, the clinical implications remain difficult to define because “cannabis use” encompasses heterogeneous products and routes, including smoked flower, vaping, concentrates, edibles, pharmaceutical cannabinoids, topical cannabidiol (CBD), and frequent co-use with tobacco or nicotine. Current evidence suggests that systemic cannabis use, particularly inhaled or heavy perioperative use, may be associated with increased surgical complications in selected populations; however, existing studies are limited by retrospective design, inconsistent exposure definitions, inadequate dose and route characterization, and confounding by tobacco use and comorbidities. Cannabinoids exert biologic effects through the endocannabinoid system, particularly CB1 and CB2 receptors, which are expressed in the central nervous system, immune cells, vasculature, and skin. These pathways influence inflammation, keratinocyte proliferation, fibroblast activity, angiogenesis, immune surveillance, pain signaling, and tissue remodeling. The net effect of cannabinoid exposure on wound healing is likely context dependent, varying based on receptor expression, wound-healing phase, route of administration, cannabinoid composition, local tissue environment, and patient-specific risk factors. Preclinical and early dermatologic literature suggests potential therapeutic roles for topical cannabinoids, especially CBD, in modulating inflammation and epithelial repair. In contrast, systemic perioperative cannabis use has been associated in several surgical cohorts with infection, delayed healing, hematoma, nonunion, and reoperation. Evidence specific to facial plastic surgery remains sparse. The most directly relevant study evaluated cannabis and tobacco use in patients undergoing operative mandibular fracture repair. Cannabis-only use was not associated with increased complications, although the cohort was small; concurrent cannabis and tobacco use was associated with higher rates of surgical site infection, facial nonunion, abscess, debridement, and malocclusion. To date, no published studies address cannabis-associated outcomes in rhinoplasty, rhytidectomy, blepharoplasty, browlift, or facial rejuvenation. This review summarizes the biologic rationale, available surgical evidence, and clinical considerations for incorporating cannabis use into individualized perioperative risk assessment in facial plastic surgery.

## 1. Introduction

Cannabis use has become increasingly prevalent over the past several decades. Population-based studies have reported substantial increases in adult cannabis use in the United States, with past-year use rising from approximately 4% in 2001 to nearly 19% by 2021 [1,2,3,4]. This trend has coincided with expanding legalization, decriminalization, and normalization of both medical and recreational cannabis use [5]. As a result, facial plastic surgeons are increasingly likely to encounter patients who use cannabis before elective aesthetic procedures, reconstructive surgery, or trauma-related operations.

Despite its growing prevalence, the perioperative relevance of cannabis remains challenging to define. The term “cannabis use” includes a broad range of exposures: inhaled flower, vaping, high-potency concentrates, edibles, tinctures, pharmaceutical cannabinoids, topical cannabidiol (CBD) preparations, and cannabis products used concurrently with tobacco or nicotine [6,7,8]. These differences matter clinically because route, dose, timing, potency, cannabinoid composition, and co-exposures may influence cardiopulmonary physiology, immune function, platelet activity, pain control, nausea, withdrawal, and wound healing. The heterogeneity of exposure is compounded by a marked rise in cannabis potency: mean delta-9-tetrahydrocannabinol (THC) concentrations in commercially available and seized cannabis have increased several-fold in recent decades, and concentrates, vaping oils, and dabbing products frequently exceed 60–90% THC [9]. Concurrent shifts toward high-potency edibles and toward daily or near-daily use may substantially amplify systemic cannabinoid exposure relative to the intermittent, lower-potency use characteristic of older cohorts, further complicating extrapolation from historical data.

Current evidence suggests an important distinction between systemic and topical cannabinoid exposure. Systemic cannabis use, especially inhaled or heavy perioperative use, may be associated with increased postoperative complications in selected surgical populations [8,10,11,12]. By contrast, topical cannabinoids, particularly CBD, have shown potential positive wound-modulating effects through anti-inflammatory, antioxidant, and tissue-remodeling mechanisms in preclinical and early clinical studies.

The evidence base in facial plastic surgery is particularly limited. There are currently no robust studies evaluating cannabis use and outcomes after common elective facial plastic procedures such as rhinoplasty, facelift, blepharoplasty, browlift, or facial fat grafting. The available facial surgery literature is largely limited to traumatic mandible fracture repair [12]. This review summarizes the current biologic and clinical evidence regarding cannabis, wound healing, and surgical outcomes, with emphasis on implications for facial plastic surgery and individualized perioperative risk stratification.

### Physiologic Mechanisms of Cannabinoid Effects on Skin and Wound Healing

Cannabis exerts many of its physiologic effects through the endocannabinoid system, a widespread neuromodulatory network composed of endogenous cannabinoids, cannabinoid receptors, and enzymes involved in endocannabinoid synthesis and degradation [13]. The two best-characterized endogenous cannabinoids are anandamide and 2-arachidonoylglycerol, which interact primarily with cannabinoid receptors CB1 and CB2 [13,14].

CB1 receptors are highly expressed in the central nervous system, including regions involved in cognition, memory, emotion, nociception, and motor control. However, CB1 receptors are also present in peripheral tissues, including vasculature, adipose tissue, and skin [13,14,15,16]. CB2 receptors are predominantly expressed in immune cells, including B lymphocytes, natural killer cells, monocytes, and macrophages, where they modulate cytokine release, immune-cell trafficking, and inflammatory tone; CB2 expression is also upregulated in keratinocytes, sebocytes, and cutaneous nerve fibers during inflammation and tissue injury [15,16]. Beyond CB1 and CB2 receptors, cannabinoids engage additional molecular targets relevant to wound biology, including transient receptor potential channels such as TRPV1, the orphan receptor GPR55, and peroxisome proliferator-activated receptors, which further diversify their downstream effects on nociception, angiogenesis, and epithelial repair [17].

Tetrahydrocannabinol is the principal psychoactive cannabinoid in cannabis and acts as a partial agonist at CB1 and CB2 receptors [7,18]. CBD has more complex pharmacology, with relatively low direct affinity for CB1 and CB2 receptors but multiple potential effects on inflammatory, nociceptive, and oxidative stress pathways [7,18,19]. These mechanistic differences are important because THC-rich inhaled cannabis, oral cannabinoid products, and topical CBD preparations should not be assumed to have equivalent effects on wound biology.

Wound healing occurs through overlapping phases of hemostasis, inflammation, proliferation, and remodeling. Cannabinoid signaling may influence each phase. During the inflammatory phase, CB2 activation can suppress T-cell proliferation, reduce macrophage cytokine production, and alter bacterial clearance. These effects may attenuate excessive inflammation but could also impair early host defense and increase infection risk in contaminated or high-risk wounds [6,19]. During the proliferative phase, cannabinoid signaling may influence keratinocyte proliferation, fibroblast activity, angiogenesis, and re-epithelialization [6,15,16,19,20,21,22]. During remodeling, cannabinoid-related effects on fibroblast activity and collagen deposition may influence scar formation and tensile strength [6,16,19]. Cannabinoid signaling also intersects with angiogenesis and vascular tone: endocannabinoids and THC can promote endothelial proliferation and vasodilation in some experimental models, whereas the combustion products of smoked cannabis—including carbon monoxide and particulate matter—may impair tissue oxygenation and microvascular perfusion in a manner mechanistically analogous to cigarette smoke [23].

The net effect is therefore unlikely to be uniformly beneficial or harmful. It overall depends on the local balance of CB1 and CB2 receptor expression, the wound-healing phase at the time of exposure, cannabinoid type, dose, route, tissue vascularity, bacterial burden, and host factors such as diabetes, immunosuppression, nutrition, tobacco use, and vascular disease [6,16,19,22]. This context dependence is especially relevant in facial plastic surgery, where outcomes depend not only on wound closure but also on scar quality, flap viability, infection prevention, hematoma avoidance, skeletal union, and aesthetic precision.

## 2. Materials and Methods

This article is a narrative clinical review structured to synthesize mechanistic and surgical evidence relevant to cannabis use and perioperative wound healing that can be correlated to facial plastic surgery. A structured narrative search was performed in MEDLINE/PubMed, Embase, and Google Scholar for studies published through 30 June 2026. Search concepts were combined using Boolean operators and included cannabis, marijuana, cannabinoid, THC, cannabidiol, CBD, intoxication, dependence, cannabis use disorder, wound, wound healing, infection, dehiscence, hematoma, nonunion, surgery, perioperative, anesthesia, pain, plastic surgery, reconstructive surgery, dermatologic surgery, burn, orthopaedic or orthopedic surgery, hand surgery, otolaryngology, facial plastic surgery, facial trauma, mandible, rhinoplasty, rhytidectomy or facelift, blepharoplasty, browlift, resurfacing, fat grafting, flap, and graft. Reference lists of relevant reviews and included articles were manually searched. Selected sources were English-language human clinical studies reporting perioperative wound healing, infection, pain, or utilization outcomes associated with cannabis exposure; reviews that synthesized clinically relevant evidence; and selected mechanistic or preclinical studies needed to explain cutaneous biology. Given the limited studies on facial plastic surgery specifically, adjacent surgical specialties were included in this expanded clinical review to discuss outcomes with plausible relevance to facial plastic surgery, including infection, hematoma, dehiscence, flap complications, nonunion, reoperation, pain, or opioid use. Excluded were non-surgical reports without a plausible perioperative connection, opinion pieces without evidentiary contribution, duplicate publications, and studies in which cannabis exposure could not be distinguished from a broader substance-use category.

**Figure 1:**
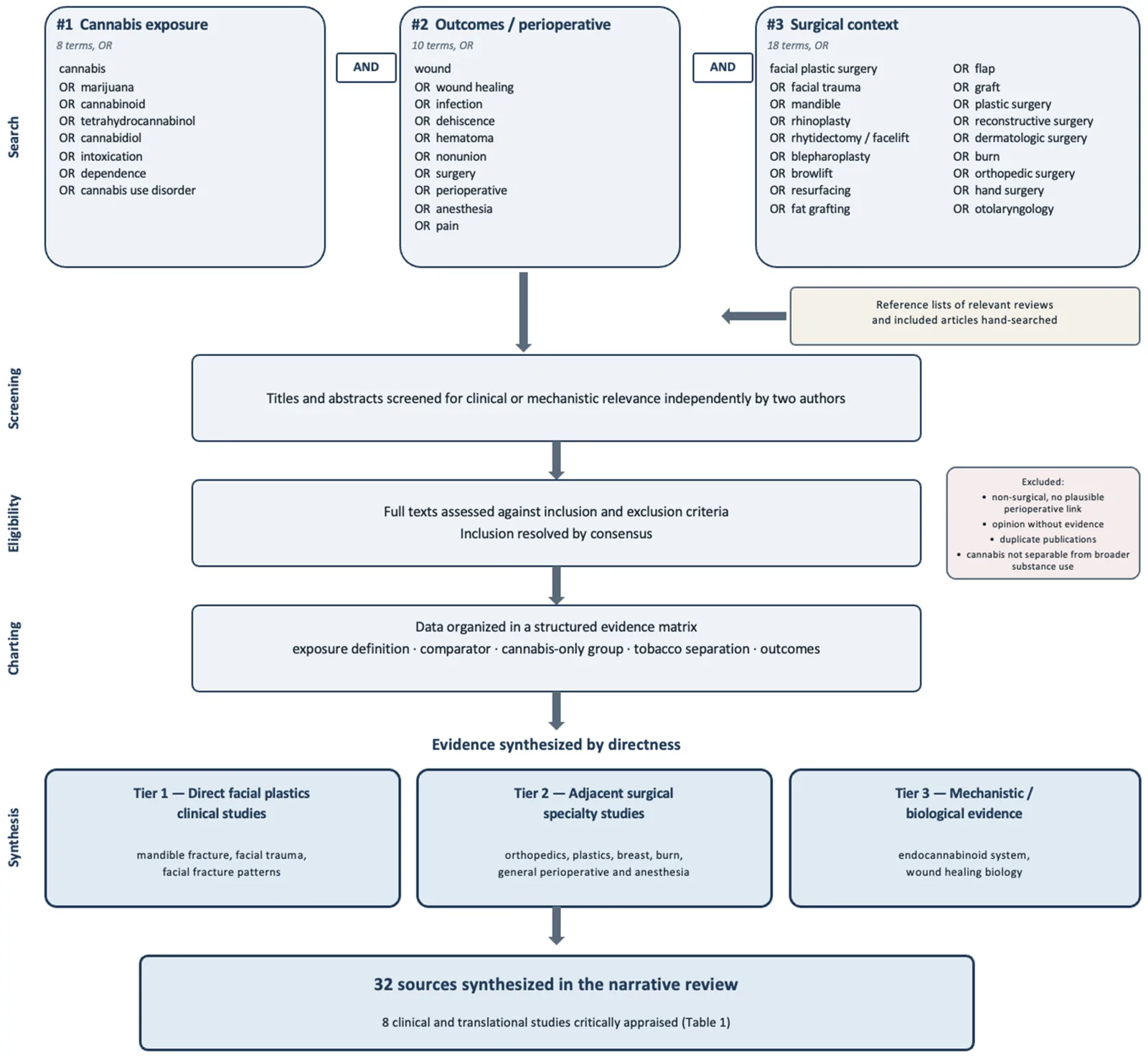
Search and selection pathway. Search concepts were combined with Boolean operators across MEDLINE/PubMed, Embase, and Google Scholar for studies published through 30 June 2026, and reference lists of relevant reviews and included articles were manually searched. Titles and abstracts were screened by two authors, full texts were assessed against the inclusion and exclusion criteria, and evidence was organized in a structured matrix and synthesized based on application.

Two authors reviewed titles and abstracts, examined potentially relevant full texts, and resolved inclusion by consensus. For each included clinical source, data was organized in a structured evidence matrix including author and year, specialty and procedure, sample size, exposure definition, comparisons, availability of a cannabis-only group, separation of tobacco or nicotine, outcome domains, principal findings, and limitations. Evidence was organized into direct facial plastics clinical evidence, adjacent surgical evidence, and mechanistic or biological evidence. Directness, exposure validity, tobacco control, study design, and outcome relevance informed the narrative appraisal in Table 1. Given the heterogeneity of study designs, exposure definitions, and outcome measures, a quantitative synthesis was not appropriate; instead, a qualitative synthesis was performed, and the key surgical studies were appraised based on design, exposure characterization, and generalizability to facial plastic surgery. The overall study-selection process is summarized in Figure 1, and the appraisal of key surgical studies in Table 1.

## 3. Clinical Evidence from Surgical Specialties

Although direct evidence in facial plastic surgery is limited, studies from orthopedic surgery, plastic surgery, and burn care provide useful context.

The orthopedic literature has raised concern that cannabis use may be associated with impaired osseous healing and increased postoperative complications. Heath et al. reviewed the orthopedic literature and reported associations between marijuana use and lower bone mineral density, increased fracture risk, delayed bone healing, increased postoperative complications, increased opioid use, and cannabinoid hyperemesis [27]. While bone healing differs from cutaneous wound healing, these findings are relevant to facial trauma, mandibular fracture repair, orthognathic procedures, and reconstructive operations involving osseous fixation.

More recent database studies have reported increased complication risk after fracture fixation. Tummala et al. found that preoperative cannabis use was associated with higher rates of infection, wound dehiscence, sepsis, hardware infection, nonunion, and reoperation after ankle open reduction and internal fixation [10]. Hamad et al. similarly reported increased surgical, medical, and psychosocial complications after lower extremity fracture fixation in cannabis users compared to non-users [24]. These findings support the concept that cannabis exposure may serve as a modifiable perioperative risk factor.

Studies within plastic surgery literature also suggests potential risk. In implant-based breast reconstruction, Al-Saghir et al. found that active marijuana use within 12 weeks of surgery was associated with higher rates of cellulitis requiring intravenous antibiotics, explantation for infection, emergency department visits, readmission, return to the operating room, and major complications [11]. Garoosi et al. likewise reported increased complications in patients with a history of cannabis and tobacco use undergoing implant-based breast reconstruction [25]. In reduction mammaplasty, Ferdousian et al. reported that perioperative marijuana use was associated with increased hematoma formation and delayed wound healing [26]. Although breast and body procedures differ from facial plastic surgery, these data are relevant because hematoma, infection, delayed healing, and reoperation are also key complications in facial aesthetic and reconstructive operations.

The burn and wound-care literature further supports caution. Nguyen et al. evaluated the impact of preexisting substance use disorders on prolonged opioid use and postoperative complications after burns, highlighting the complex relationship between substance use, postoperative pain, wound recovery, and health care utilization [28]. Reviews by Parikh et al. and Niyangoda et al. emphasize that cannabinoid effects on wound healing remain incompletely characterized, with much of the supportive evidence derived largely from preclinical and in vitro models, small case series, and heterogeneous product formulations rather than adequately powered clinical trials [6,29]. Collectively, these cross-specialty data establish biologic plausibility for both beneficial and harmful cannabinoid effects on healing, but they do not yet permit procedure-specific risk quantification in facial plastic surgery.

The broader otolaryngology and anesthesiology literature adds further context with direct relevance for facial surgeons. Broad reviews of surgery-related considerations in patients who use cannabis highlight potential effects on airway reactivity, anesthetic and analgesic requirements, hemodynamic lability, and postoperative nausea and vomiting, alongside wound-healing concerns [8]. Large database and cohort analyses have likewise associated non-medical cannabis use with increased post-procedural healthcare utilization across surgical and interventional populations [2]. Because these physiologic and perioperative effects converge on the same complications that affect facial aesthetic and reconstructive results—bleeding, hematoma, wound disruption, and infection—these findings reinforce the relevance of cross-specialty evidence to facial plastic surgery even in the absence of procedure-specific data.

## 4. Topical Cannabinoids and Cutaneous Wound Healing

Topical cannabinoids should be considered separately from smoked, vaped, or ingested cannabis. Dermatologic and preclinical literature suggests that topical cannabinoids may have anti-inflammatory, analgesic, antipruritic, antioxidant, and wound-modulating effects [6,19]. Kong et al. reviewed the potential role of cannabinoids in dermatologic surgery, including their relevance to inflammation, pain, and wound modulation [30]. Shah et al. reviewed cutaneous wound healing and CBD, highlighting experimental evidence that CBD may influence inflammation, oxidative stress, epithelialization, and tissue repair [31].

However, these findings should not be overinterpreted. Most clinical evidence for topical cannabinoids remains preliminary, and commercially available products vary substantially in CBD concentration, THC contamination, excipients, purity, and regulatory oversight. In facial plastic surgery, scar quality, pigmentation, infection risk, and wound tensile strength are critical outcomes; therefore, topical cannabinoids should be considered investigational adjuncts rather than routine postoperative wound-care agents. Their use should be approached cautiously, especially near fresh incisions, grafts, flaps, implants, or mucosal wounds.

## 5. Cannabis Use in Facial Plastic Surgery

Evidence specific to facial plastic surgery remains sparse. To our knowledge, the most directly relevant published clinical study is the analysis by Yoon et al. evaluating postoperative complications related to cannabis and tobacco use after operative repair of mandibular facial fractures [12]. Using the PearlDiver database, Yoon et al. identified 8288 patients who underwent mandible fracture repair between 2010 and 2020. Patients were stratified into four groups: no cannabis or tobacco use, cannabis-only use, tobacco-only use, and combined cannabis and tobacco use.

The cannabis-only cohort was small, comprising 72 patients, or 0.86% of the study population. By comparison, 914 patients had used both cannabis and tobacco, 3236 used tobacco only, and 4066 served as controls. Outcomes included surgical site infection, facial nonunion, facial abscess, debridement, and malocclusion [12].

Cannabis-only use was not associated with a statistically significant increase in postoperative complications compared with controls. However, concurrent cannabis and tobacco use was associated with increased risk of surgical site infection, facial nonunion, facial abscess, need for debridement, and malocclusion. Tobacco-only use was also associated with increased complications [12]. These findings suggest that tobacco remains a major confounder and potential effect modifier in studies of cannabis-related surgical risk.

Several limitations should temper interpretations. First, the cannabis-only cohort was very small relative to the expected prevalence of cannabis use in the general population. Second, database studies rely on diagnosis and procedure coding, which may undercapture cannabis exposure, tobacco exposure, frequency of use, route of use, and dose. Third, the study could not distinguish smoked cannabis from edibles, vaping, concentrates, or medical cannabinoid products. Fourth, cannabis and tobacco exposure may have been incompletely documented, especially in trauma settings. Finally, mandibular fracture repair involves contaminated oral flora, rigid fixation, occlusion, trauma severity, and bone healing, which may not generalize to elective facial aesthetic surgery.

There are no high-quality studies evaluating cannabis exposure and outcomes after rhinoplasty, rhytidectomy, blepharoplasty, browlift, facial resurfacing, facial fat grafting, or elective facial reconstruction. This represents an important gap because these procedures are highly sensitive to hematoma, edema, bruising, infection, flap perfusion, scar maturation, and patient adherence to postoperative instructions.

Although direct data are lacking, the mechanisms and cross-specialty findings summarized above map onto specific facial plastic procedures in predictable ways, producing a structured framework for individualized counseling. Concerns regarding impaired osseous healing and nonunion—drawn from orthopedic fixation and mandibular fracture data—are most applicable to nasal and orthognathic osteotomies, septorhinoplasty with bony work, and craniomaxillofacial fracture repair [10,12,27]. Findings of flap compromise, hematoma, and delayed healing from implant-based breast reconstruction and reduction mammaplasty are conceptually relevant to cervicofacial rhytidectomy, forehead and paramedian forehead flaps, and other local flap reconstructions, where perfusion and hematoma avoidance are critical [11,25,26]. Infection and explantation outcomes from alloplastic breast reconstruction parallel the risks of alloplastic facial implants used in chin, malar, and dorsal nasal augmentation [11,25]. Cannabinoid effects on angiogenesis and re-epithelialization are relevant to flap and graft survival in addition to laser and chemical resurfacing outcomes including epithelial repair and scar quality [6,19,31]. Addressing the procedure-specific evidence provides actionable corollaries for facial plastic surgery.

## 6. Perioperative Pain, Opioid Use, and Recovery

Pain management is another important interface between cannabis use and wound recovery. Many patients use cannabis for acute or chronic pain, anxiety, insomnia, nausea, or perceived opioid reduction. However, evidence supporting cannabinoids for postoperative pain remains inconsistent. Stephens et al. concluded that the evidence for oral and topical CBD in plastic surgery pain management is inconclusive [32]. Hsu et al. similarly concluded in a broad JAMA review that the evidence remains insufficient for most therapeutic uses of cannabis and cannabinoids [7].

Poorly controlled pain may impair sleep, nutrition, mobility, wound care, and adherence to postoperative restrictions. Conversely, heavy cannabis use may be associated with increased anesthetic requirements, postoperative nausea and vomiting, cannabinoid hyperemesis, increased opioid requirements, and increased health care utilization [7,8,27,32]. These issues are particularly relevant in facial plastic surgery, where coughing, retching, hypertension, nonadherence, and agitation may increase the risk of bleeding, hematoma, wound disruption, or graft compromise.

## 7. Personalized-Medicine Approach to Perioperative Cannabis Risk

A tailored, personalized-medicine approach is well suited to cannabis-related perioperative assessment given that the risks are not uniform across patients or procedures. Rather than treating cannabis exposure as a binary variable, a detailed history should be obtained and interpreted in the context of the planned surgery and the patient’s comorbidities.

Preoperative screening should include product type, route of administration, frequency, dose or potency, timing of last use, duration of use, medical versus recreational use, history of cannabis use disorder, prior withdrawal symptoms, hyperemesis, and concurrent tobacco or nicotine use [8]. Surgeons should also ask specifically about vaping, high-potency concentrates, edibles, CBD products, and topical preparations because many patients may not volunteer these details unless asked directly. Consistent with a personalized-medicine framework, this variability argues for individualized rather than categorical risk assignment, and for eventual incorporation of objective exposure measures—such as quantitative serum or urinary THC and metabolite levels—to complement self-reported history and reduce exposure misclassification.

Higher-risk features include heavy or daily use, inhaled cannabis use, high-potency THC products, recent intoxication, cannabis use disorder, concurrent tobacco or nicotine use, cardiopulmonary disease, diabetes, immunosuppression, malnutrition, prior wound complications, radiation exposure, flap-based reconstruction, implant use, skeletal fixation, and contaminated traumatic wounds [6,8,10,11,12,24,27]. In patients with these features, cannabis use should be discussed as a potentially modifiable perioperative risk factor, similar to tobacco use, while acknowledging that the evidence remains incomplete.

Perioperative counseling should be individualized. Patients should be advised not to present for elective surgery acutely intoxicated. For inhaled cannabis users, cessation or reduction before surgery may be reasonable, particularly for procedures where bleeding, pulmonary irritation, coughing, flap perfusion, infection, or wound healing are major concerns. Patients using cannabis medically should be managed collaboratively with anesthesia, primary care, pain medicine, or other involved specialties. Abrupt cessation in heavy users may precipitate withdrawal, anxiety, insomnia, irritability, appetite changes, or increased pain, all of which may affect recovery.

## 8. Clinical Implications for Facial Plastic Surgeons

For facial plastic surgeons, the current evidence supports practical, risk-based management rather than categorical exclusion of all cannabis users from surgery. Key considerations include:

1. **Screen routinely and specifically.** Cannabis exposure should be documented with the same level of detail as tobacco, alcohol, anticoagulants, supplements, and prescription medications.
2. **Differentiate route and product.** Smoked cannabis, vaping, edibles, concentrates, medical cannabinoids, and topical CBD should not be treated as equivalent exposures.
3. **Identify tobacco and nicotine co-use.** Studies suggest that the cannabis-tobacco interaction can be synergistic and amplify adverse effects on wound healing [8,26].
4. **Assess procedure-specific risk.** Cannabis-related concerns may be more consequential in operations involving large skin flaps, implants, grafts, bone fixation, contaminated wounds, or high hematoma risk.
5. **Coordinate with anesthesia.** Cannabis use may affect airway reactivity, anesthetic dosing, hemodynamics, postoperative nausea, pain control, and emergence.
6. **Counsel transparently.** Patients should be informed that the evidence is evolving, but systemic cannabis use may plausibly increase risks of infection, delayed healing, hematoma, nonunion, reoperation, and postoperative symptom burden in selected settings.

## 9. Conclusions

Cannabis may influence wound healing through immune, inflammatory, vascular, microbial, analgesic, and behavioral pathways. Its clinical significance in facial plastic surgery remains incompletely defined because cannabis exposure is heterogeneous and frequently confounded by tobacco or nicotine use. The preclinical and dermatologic literature suggests that topical cannabinoids, especially CBD, may have positive wound-modulating potential, but the current evidence is insufficient to recommend routine cannabinoid-based postoperative wound therapy.

By contrast, systemic perioperative cannabis use—particularly inhaled, heavy, or concurrent cannabis and tobacco use—has been associated with increased complications in several surgical populations. In facial plastic surgery, the available evidence is limited primarily to mandibular fracture repair, where cannabis-only use was not statistically associated with increased complications; however, this finding is limited by the very small cohort size. Combined cannabis and tobacco use was associated with higher rates of infection, facial nonunion, abscess, debridement, and malocclusion.

Future studies should prospectively evaluate cannabis exposure in facial plastic surgery using standardized definitions that include route, frequency, dose, potency, timing of last use, indication, and tobacco or nicotine co-use. Outcomes should include infection, hematoma, dehiscence, delayed healing, scar quality, flap or graft compromise, skeletal union, pain control, opioid use, patient-reported outcomes, and revision surgery. Until such data are available, facial plastic surgeons should incorporate cannabis use into individualized perioperative risk assessment, document exposure carefully, counsel patients regarding plausible risks, and coordinate perioperative care with anesthesia and other relevant clinicians.

Several concrete research priorities emerge from this review. First, prospective multi-institutional registries and cohort studies within facial plastic surgery are needed that capture cannabis exposure with the granularity that has been reported for tobacco use studies, including product type, route, potency, cumulative dose, frequency, indication, and timing of last use. Second, biomarker-based exposure verification such as quantitative serum or urinary THC and metabolite concentrations could reduce the misclassification that limits current database studies and enable dose–response analysis. Third, procedure-specific outcomes should be standardized across studies, incorporating validated scar-assessment scales, patient-reported outcome measures, objective flap and graft perfusion metrics, and consistent infection and hematoma definitions. Fourth, adequately powered randomized trials of standardized, pharmaceutical-grade topical cannabidiol are warranted to determine whether the wound-modulating effects observed in preclinical models translate into improved scar quality or reduced inflammation after facial plastics procedures. Finally, studies should explicitly model the cannabis–tobacco interaction and test whether structured preoperative cessation or reduction protocols, analogous to established smoking-cessation pathways, measurably reduce complications in cannabis users.

## Figures and Tables

**Table 1:** Appraisal of key surgical studies informing cannabis-related perioperative risk, including design, exposure characterization, principal findings, methodological limitations, and generalizability to facial plastic surgery. BMD, bone mineral density; DB, database; ED, emergency department; FPS, facial plastic surgery; IV, intravenous; ORIF, open reduction and internal fixation; PSM, propensity score matching; SSI, surgical site infection; SUD, substance use disorder.

Study [Ref]	Specialty	Design	Cannabis Exposure	Key Findings	Quality/ Limitations	Generalizability to Facial Plastic Surgery
Yoon 2023—mandibular fracture repair [12]	Facial plastic surgery	Retrospective claims DB; *N* = 8288; cannabis-only (*n* = 72)	Cannabis-only vs. tobacco-only vs. combined; route/dose/potency not captured	Cannabis-only: no significant increase in complications; cannabis + tobacco: higher SSI, facial nonunion, abscess, debridement, malocclusion	Very small cannabis-only cohort; coding-based exposure	High—the only FPS-specific study, but trauma context rather than elective or aesthetic surgical conditions
Tummala 2026—ankle ORIF [10]	Orthopedic surgery	Retrospective claims DB; large cohort	Cannabis use vs. no cannabis use; PSM includes tobacco use	Higher infection, dehiscence, sepsis, hardware infection, nonunion, reoperation	Coding-based; no dose data	Moderate—bone healing relevant to FPS osteotomies and facial fracture plating
Hamad 2026—lower-extremity fracture fixation [24]	Orthopedic surgery	Retrospective cohort	Cannabis-only; Nicotine-only; Cannabis and nicotine	Higher rates of surgical, medical, and psychosocial complications for each exposure; concurrent tobacco and cannabis use does not have additional risk compared to cannabis-only	Separates exposures; Coding-based	Moderate—supports cannabis–tobacco synergy central to FPS counseling
Al-Saghir 2024—implant-based breast reconstruction [11]	Breast/plastic surgery	Retrospective cohort	Active marijuana within 12 weeks	Higher cellulitis rates requiring IV antibiotics, explantation, ED visits, readmission, reoperation, major complications	Single-center study; self-reported exposure	Moderate—implant infection/explantation parallels facial alloplastic implants
Garoosi 2023—implant-based breast reconstruction [25]	Breast/plastic surgery	Retrospective cohort	Cannabis and tobacco history	Increased complications with combined exposure	Only combined exposure—does not separate tobacco from marijuana	Low—reinforces combined-use risk
Ferdousian 2026—reduction mammaplasty [26]	Breast/plastic surgery	Retrospective cohort	General marijuana use	Increased hematoma and delayed wound healing	Retrospective; body-site differences	Moderate—hematoma and delayed healing
Heath 2022—orthopedics review [27]	Orthopedic surgery	Narrative review	General marijuana use	Lower BMD, higher fracture risk, delayed bone healing, more complications and opioid use, hyperemesis	Narrative; heterogeneous primary data	Moderate—bone-health relevance to the facial skeleton
Nguyen 2025—burn/wound care [28]	Burn/wound care	Retrospective analysis	Preexisting substance use disorder	Prolonged opioid use, more complications and health-care utilization	SUD is broader than cannabis alone; confounding	Low—not cannabis specific; pain and recovery relevance to perioperative care

## Data Availability

No new data were created or analyzed in this study. Data sharing is not applicable to this article.
